# Case report: Unusual patient with dermatomyositis associated with SARS-CoV-2 infection

**DOI:** 10.3389/fneur.2023.1122475

**Published:** 2023-05-19

**Authors:** Joanna Niedzielska, Monika Chaszczewska-Markowska, Justyna Chojdak-Łukasiewicz, Jakub Berezowski, Seema Kalra, Przemysław Jazwiec

**Affiliations:** ^1^Department of Neurology, Specialist Medical Center in Polanica Zdrój, Polanica-Zdrój, Poland; ^2^Laboratory of Clinical Immunogenetics and Pharmacogenetics, Ludwik Hirszfeld Institute of Immunology and Experimental Therapy, Polish Academy of Sciences, Wroclaw, Poland; ^3^Department of Neurology, Faculty of Medicine, Wroclaw Medical University, Wroclaw, Poland; ^4^Department of Administration, Jan Mikulicz Radecki University Teaching Hospital in Wroclaw, Wroclaw, Poland; ^5^Department of Neurology, Royal Stoke University Hospital, University Hospitals of North Midlands NHS Trust, Stoke-on-Trent, United Kingdom; ^6^Department of Imaging Diagnostics, Specialist Medical Center in Polanica Zdrój, Polanica-Zdrój, Poland

**Keywords:** SARS-CoV-2, COVID-19, dermatomyositis, extrapulmonary manifestation, neurological complication

## Abstract

The severe acute respiratory syndrome coronavirus 2 (SARS-CoV-2) outbreak is a major challenge for clinicians. SARS-CoV-2 infection results in coronavirus disease 2019 (COVID-19), and it is best known for its respiratory symptoms. It can also result in several extrapulmonary manifestations such as neurological complications potentially experienced during the course of COVID-19. The association of dermatomyositis (DM) with COVID-19 pathogenesis has not been well-studied. This study aimed to present a previously healthy 37-year-old man, a soldier by profession, with symptoms of DM on the 4th day from the onset of COVID-19. The patient presented DM symptoms with both skin and muscle manifestations. The patient suffered from cough, fever, and fatigue to begin with, and reverse-transcription polymerase chain reaction (RT-PCR) reported positive for SARS-CoV-2 infection. The laboratory findings showed, intra alia, elevated muscle enzymes CK 8253 U/l (N: <145 U/l), a positive test for myositis-specific autoantibodies (anti-Mi-2), electrodiagnostic tests exhibited features of myopathy, with the presence of muscle and skin symptoms. The patient improved with corticosteroids and immunosuppressive agent therapy. In summary, the association between COVID-19 and the development of multi-system autoimmune disorders such as DM remains unclear. Nevertheless, viral infections such as SARS-CoV-2 may likely serve as a trigger.

## 1. Introduction

Dermatomyositis (DM) is one of the idiopathic inflammatory myopathies. It is relatively rare with an incidence rate of 7.98 per 1 million in the adult population per year, predominately women (1). Idiopathic inflammatory myopathy appears in adults between the ages of 40 and 60 years or in children between the ages of 5 and 15 years as juvenile DM (JDM).

The diagnosis of DM depends upon the presence of characteristic clinical and laboratory findings, including symmetric proximal skeletal muscle weakness, elevated muscle enzymes, and skin symptoms (2). Occasionally, internal organs are affected as in this case (3). The diagnosis of classic DM does not require a tissue biopsy in patients with clinical and laboratory findings that are particularly characteristic of this disorder. Such characteristics principally include symmetric proximal muscle weakness in the setting of a marked elevation of muscle enzymes, where evidence suggesting an alternative diagnosis is lacking (4).

Various classification criteria for DM have been suggested by a number of investigators. Currently, the most recognized are The European League Against Rheumatism/American College of Rheumatology (EULAR/ACR) classification criteria (5). According to the proposed classification, the diagnosis of DM is probable if the patient scores >6.7 points with a muscle biopsy or >5.5 points without a muscle biopsy. The diagnosis of DM is certain if the patient scores >8.7 and >7.5 points, respectively, according to the score assigned to each symptom (6).

The etiology of DM remains unknown; some studies have reported an association with histocompatibility antigens, environmental agents (e.g., viruses, drugs), and autoimmunity (7). The severe acute respiratory syndrome coronavirus 2 (SARS-CoV-2) infection can break immune tolerance and trigger autoimmune responses, and it is also likely to induce clinical autoimmunity (8). Indeed, numerous reports have confirmed the development of autoimmune diseases after SARS-CoV-2 infection, e.g., Guillain–Barré syndrome, idiopathic thrombocytopenic purpura, autoimmune hemolytic anemia (9), autoimmune thyroiditis (10), and systemic lupus erythematosus (11). Other autoimmune diseases induced by SARS-CoV-2 may be reported in future.

Dermatomyositis (DM) is a systemic disorder, and the skin and muscles are the most commonly affected organs. However, patients may also present themselves with many other dysfunctions including arthralgia, arthritis, esophageal disease, and cardiopulmonary dysfunction. Notably, DM has been linked to cancer. The cancer-associated disease is more commonly found in older patients (12). According to Louis et al. (1), DM has been linked to internal malignancy in somewhere between 15% and 20%. Without a doubt, DM must be distinguished from other conditions that cause muscle weakness, with or without muscle enzyme elevation.

Treatment recommendations for patients with DM are based on disease severity and the presence of systemic symptoms. Management of DM is usually based on systemic corticosteroids with or without an immunosuppressive agent (13). Pharmacological agents include methotrexate, azathioprine, mycophenolate mofetil, cyclophosphamide, hydroxychloroquine, and calcineurin inhibitors. Intravenous immunoglobulin (IVIG) is effective in the treatment of refractory DM (14). There are new and emerging therapeutics for DM, but further studies and observations are needed.

Since the SARS-CoV-2 outbreak in 2020, an increased number of DM cases have been reported (14). However, the pathogenesis of coronavirus disease 2019 (COVID-19) related to DM has not been well-studied. There are a few hypotheses that may explain its existence. This study presents a case report of a previously healthy 37-year-old man, a soldier by profession, with symptoms of DM on the 4th day from the onset of COVID-19.

## 2. Case report

This study was carried out in accordance with the guidelines of the Declaration of Helsinki and Good Clinical Practice. The study was approved by the Local Bioethics Committee of the Wroclaw Medical University (approval no. KB−552/2021). Informed consent was obtained from the subject involved in the study. The patient in our case report was a 37-year-old healthy man and a soldier by profession. Apart from one operation undergone for lumbosacral discopathy, the patient had no significant medical history to report.

During an episode of SARS-CoV-2 infection, the patient developed the first symptoms of DM. The diagnosis of DM is based on the abovementioned EULAR/ACR criteria and the presence of at least one of the highly characteristic skin lesions. The score of the patient discussed in this study was 12.8 points, according to the score assigned to each symptom. The patient declared neither injury nor any use of new drugs or other substances in the time preceding the onset of symptoms. The patient contracted COVID-19 in February 2021. SARS-CoV-2 infection was confirmed by a PCR test on 02 February 2021. The disease first presented itself as a worsening mood alongside general infection symptoms. After 4 days, a loss of taste and smell and a sudden deterioration of locomotive functions—including gait disturbances, followed alongside difficulties with walking upstairs, getting up from a sitting position, raising the hands, and moving on a flat surface. The locomotive symptoms intensified in the following weeks with the addition of falls and issues with swallowing. The patient did not present any serious respiratory symptoms. The patient has been hospitalized several times in the Hospital Emergency, Neurology, and Rheumatology Departments during the period between May 2021 and November 2021. Physical examination revealed generalized muscle weakness, predominantly in proximal muscles with the addition of localized pain in those areas, head drop, and significant restriction of joint mobility due to pain, mainly in the shoulder joints. Skin changes were present in the form of erythema and the swelling of the eyelid area (“heliotrope rash”), red-blue streaked lesions on the neck, chest, and back (“V-sign”), red discoloration on the scalp, and discoloration above the metacarpophalangeal joints (Gottron's papules).

The patient reported polyarticular joint pain, severe muscle pain, and a burning sensation in the muscles in conjunction with difficulty in swallowing. At the most severe periods, during the course of the disease, the patient required the care of other people. Laboratory tests showed elevated levels of CK8253 U/l, AST 276 U/l, ALT 167 U/l, CKMB mass >300 ng/ml Troponin T 578.9 ng/l (Table 1). Moreover, antinuclear antibodies were detected in blood serum: ANA in the measure 1:5120 and p/Mi-2 (Table 1). Anti-Mi-2 is one of the five DM-specific antibodies, and their presence is associated with a more severe onset of the disease, a better treatment response, and a better prognosis (15).

**Table 1:** Laboratory results in 3 and 4 months from the onset of COVID-19 and DM.

**Test**	**Result (reference range)**
**Laboratory results after 3 months from onset of COVID-19**	
**and DM**	
Complete blood count	Normal
CK U/l	8253 (< 145)
ALT U/l	167 (< 50)
AST U/l	276 (< 35)
Bilirubin	Normal
Amylase	Normal
Albumin	Normal
CKMB-mass (ng/ml)	>300 (< 6.22)
Troponin T (ng/l)	578.9 (< 14)
NT-proBNP pg/ml	Normal
hs-CRP (mg/l)	6.6 (< 5)
D-dimer (ug/ml)	0.81 (0.0–0.5)
Electrolytes	Normal
Creatinine mg/dl	0.5 (0.72-1.18)
eGFR (mL/min/1.73m2)	>90
Glucose	Normal
Rheumatic factor	Negative
Urinalysis	Normal
**Laboratory results after 4 months from onset of COVID-19**	
**and DM**	
Anti-Mi-2	Positive
ANA titer	1:5120
Anti-CCP U/ml	Negative
Antigen CA 19-9 U/ml	Negative
CEA	Negative
PSA	Negative
Hepatitis panel	Negative
Complement system C3, C4	Negative
ALP	Normal
HIV-1 and−2 Ag/Ab	Negative
TSH	Normal
ESR mm/h	Normal
Troponin I	Normal
Vitamin D3 ng/ml	12.3 (30–80)
Ca and Mg level	Normal
Protein electrophoresis	Normal

After 4 months from the disease onset, extended diagnostics were performed in the Rheumatology Department.

After 9 months from the disease onset, all the laboratory parameters normalized.

CK, creatine kinase; ALT, alanine aminotransferase; AST, aspartate transaminase; CK-MB, creatine kinase myocardial band; NT-proBNP; N-terminal pro b-type natriuretic peptide; hs-CRP, high-sensitivity C-reactive protein; eGFR, estimated glomerular filtration rate; Anti-Mi-2, Mi-2 antigen; ANA, antinuclear antibodies; Anti-CCP, cyclic citrullinated peptide antibodies; Antigen CA, cancer antigen; CEA, carcinoembryonic antigen; PSA, prostate-specific antigen; ALP, alkaline phosphatase; HIV 1 and 2 Ag/Ab, human immunodeficiency virus 1 and 2 antigen/antibody; TSH, thyroid-stimulating hormone; ESR; erythrocyte sedimentation rate; Ca, calcium; Mg, magnesium.

Electromyography (EMG) tests revealed disorders corresponding to myogenic muscle damage. Primary muscle damage, proximal with the presence of fibrillation and positive free waves in the resting record—suggests inflammatory myopathy (Supplementary Table 1).

Subsequently, diagnostics were extended to include a histopathological examination of skin and muscle biopsy, the results of which showed no feature characteristics of DM. A diagnosis of DM was ultimately established based on the clinical picture, and the results of laboratory and electrophysiological tests. Treatment with glucocorticosteroids was initiated: initially, prednisone at a dose of 40 mg/day, then 60 mg/day, and subsequently, an immunosuppressive drug was added—methotrexate at a dose of 25 mg/week subcutaneously.

Further testing revealed the normal cerebrospinal fluid after examination and negative Pandy and Nonne-Apelt tests. A head MRI with contrast enhancement showed the presence of a pineal gland cyst sized 11 x 9 x 9 mm. With this aside, brain imaging was normal, without pathological contrast enhancement. The myocardium MRI showed non-ischemic (post-inflammatory) changes in the lateral wall. There was no pericardial effusion. Lake Louise criteria for the diagnosis of an active inflammatory process in the heart muscle are not met (16).

The echocardiogram results were normal. Additionally, the patient underwent testing to exclude the presence of neoplastic changes, e.g., head, chest, and abdominal cavity CT, all of which returned no changes from normal. Endoscopic examinations of the gastrointestinal tract (gastroscopy and colonoscopy) and histopathological biopsies taken from the gastric mucosa and the large intestine did not reveal any changes suspected of being cancerous.

In the months following the disease onset, there was a downward trend in CK, CK-MB, and transaminase activity, up to the complete normalization of parameters in laboratory tests in November 2021. The patient's health condition started showing signs of improvement approximately 6 months after falling ill, and it was during this time that they initially visited the neurological clinic.

Weight loss of ~30 kg was reported by the patient since the disease onset in February 2021. Persistent generalized weakness, muscle pain, dysphagia, joint pain, memory, and concentration disorders were also reported. Upon physical examination, the following was observed; first, the patient was emaciated, there were no disturbances in the innervation of the cranial nerves (including symmetrical palatopharyngeal reflexes), muscle mass was reduced, 4-limb flaccid paresis was present; the upper and lower extremities were graded at 2/5 on the Medical Research Council (MRC) muscle scale, and significant weakening of the muscles of the shoulder, hip, and neck muscles was also observed. Further observations included a lack of deep reflexes in three limbs, hypoesthesia of the anterior and lateral surfaces of the right thigh, and standing from a supine position was achieved using the Gowers sign. Walking independently was very unsteady. There were no meningeal and pyramidal symptoms. A rash on the scalp and discoloration on the metacarpophalangeal and interphalangeal joints were present.

**Figure 1:**
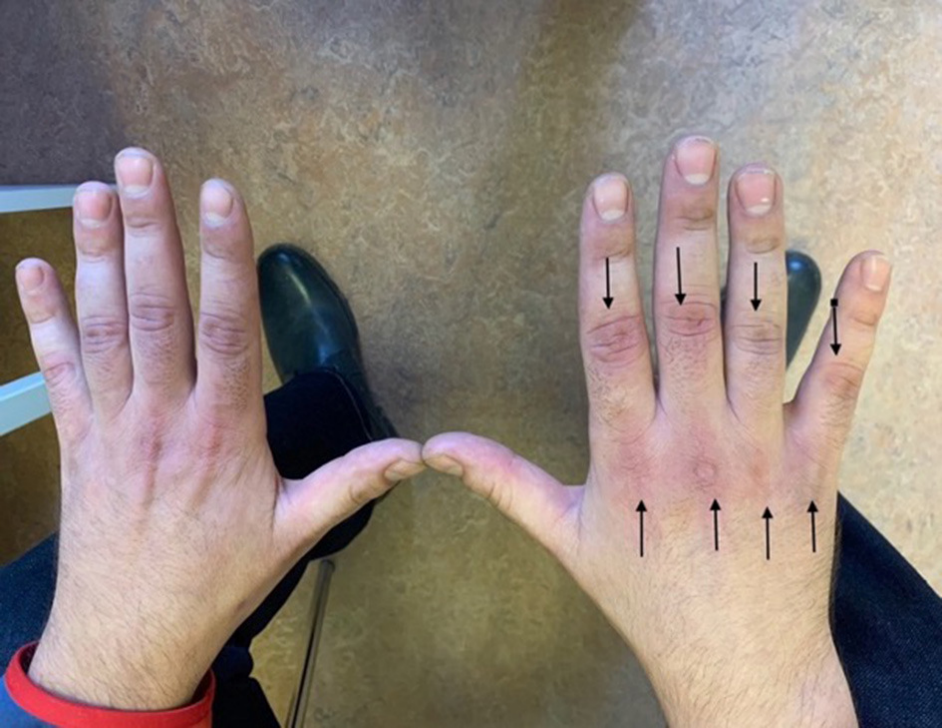
Gottron's papules—9 months from the DM onset.

During therapy, prednisone at a dose of 60 mg/day and methotrexate at a dose of 25 mg/week were administered. The patient received a referral to continue physical rehabilitation. The next follow-up visit took place 9 months after the disease onset (in December 2021), and upon physical examination, there was a significant improvement in the strength of the shoulder and pelvic girdles. In the upper and lower left limbs, muscle scale was graded at 4/5, and the lower right limb was 3/5. Neck muscle strength presented as normal, and the Gowers sign was no longer used when standing from the supine position. Gait was independent and efficient. Discoloration and ulceration of the skin over Gottron's papules were still visible above the metacarpophalangeal and interphalangeal joints during the healing phase (Figure 1). Painful hard lumps in the subcutaneous tissue of the shoulder area were present alongside the side effects of chronic steroid therapy (e.g., cushingoid appearance and parchment skin).

The patient reported a significant improvement in wellbeing, alongside their weight increasing by approximately 10 kg. The patient is also undergoing systematic rehabilitation. Prednisone was tapered down to 35 mg/week, and methotrexate remained at 25 mg/week.

**Figure 2:**
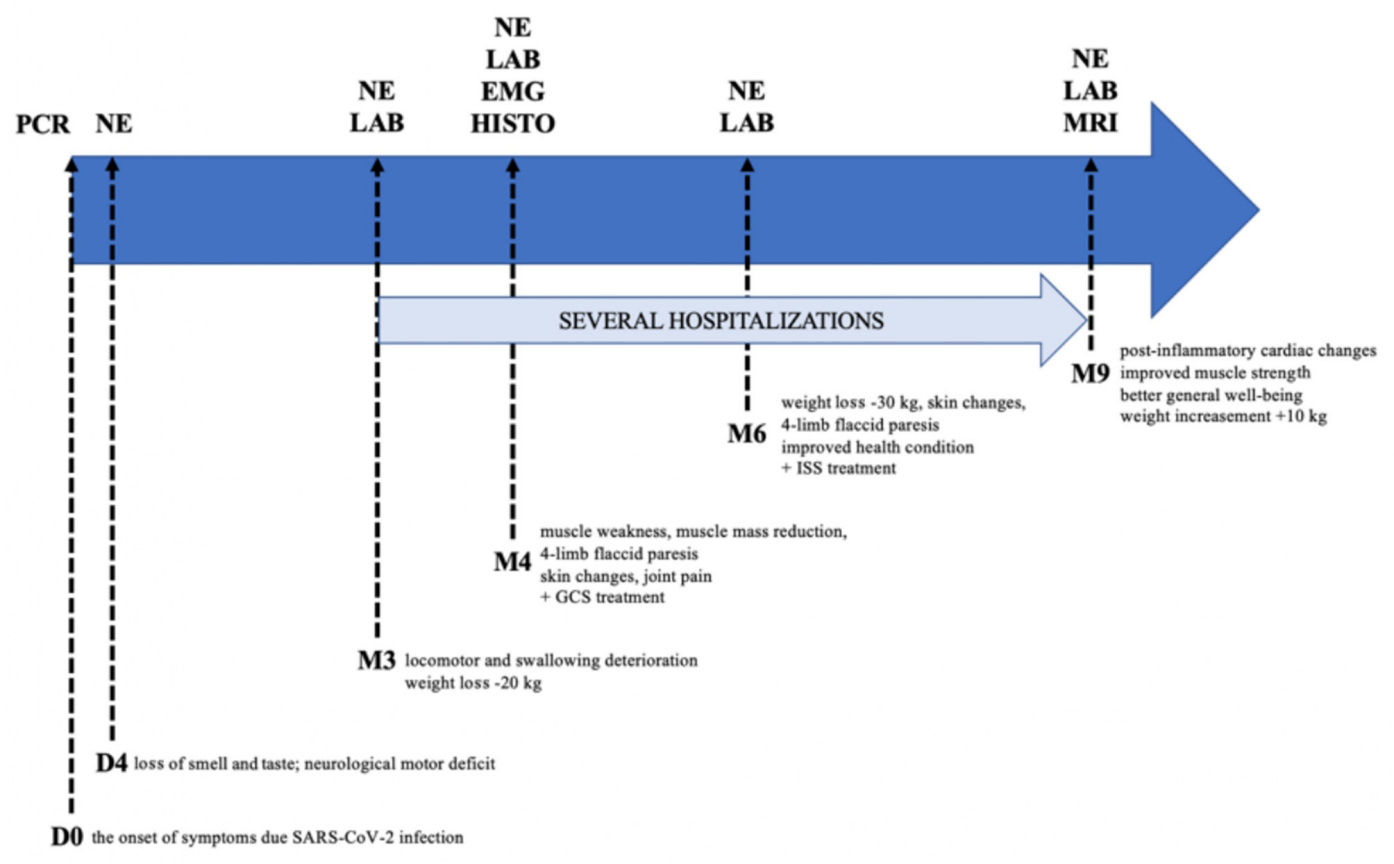
Graphical study timeline. PCR, polymerase chain reaction; NE, neurological examination; LAB, laboratory testing; EMG, electromyography; HISTO, histopathological examination; MRI, magnetic resonance imaging; GCS, glucocorticosteroids; ISS, immunosuppressants; D, day; M, month.

The patient attempted to return to work in his role as a professional soldier after 8 months; however, his health prevented it. Therefore, the patient was on sick leave for many months. He is currently under the care of a neurologist, cardiologist, and rheumatologist. A detailed study timeline is presented in Table 2 and Figure 2.

**Table 2:** Detailed study timeline with the characteristics of patients' condition including diagnostic and treatment procedures.

**Time**	**Characteristics**	
Day 0 Onset of symptoms	° Cough, fever, and fatigue ° PCR reported positive for SARS-CoV-2	
Day 4	° A loss of taste, and smell, and a sudden deterioration of locomotive functions - including gait disturbances followed, alongside difficulties with walking upstairs, getting up from a sitting position, raising the hands, and moving on a flat surface	
Week 1-12	° The locomotive symptoms intensified with the addition of falls and issues with swallowing	
Month 3	Months 3-9 The patient has been hospitalized several times	° Laboratory results: CK U/l = 8253, CKMB-mass (ng/ml) = >300, ALT U/l = 167, AST U/l = 276 ° Weight loss of - 20 kg ° Generalized muscle weakness and deterioration of locomotive symptoms
Month 4		° -Anty-Mi-2 POSITIVE; ANA titer 1:5120; CK U/l=2757 U/l ° 4-limb flaccid paresis, generalized muscle weakness, predominantly in proximal muscles, head drop ° Significant restriction of joint mobility due to pain ° Skin changes ° Electromyography (EMG) tests revealed disorders corresponding to myogenic muscle damage. Detailed findings on the attached image. ° Treatment with glucocorticosteroids was initiated. ° No features of DM in a histopatological examination. ° Exclude the presence of neoplastic change ° The muscle mass was reduced
Month 6		° An immunosuppressive drug was added to treatment ° Weight loss of 30 kg ° The muscle mass was reduced, 4-limb flaccid paresis was present still ° The upper and lower extremities were graded at 2 / 5 on the Medical Research Council (MRC) muscle scale. Walking independently was very unsteady ° Skin changes ° There was a downward trend in CK, CK-MB, and transaminase activity ° The patient's health condition began to improve
Month 9		° The myocardium MRI showed non-ischemic (post-inflammatory) changes in the lateral wall ° There was a significant improvement in the strength of the shoulder and pelvic girdles, in the upper and lower left limbs muscle scale was graded at 4/5 and the lower right limb was 3/5 ° The complete normalization of parameters in laboratory tests ° The patient reported an improvement in well-being, their weight increasing by around 10 kg ° Necessary regular physical rehabilitation

## 3. Discussion

Since the outbreak of COVID-19, an increased number of inflammatory myopathies (including DM) have been reported. The pathogenesis of COVID-19-related DM has not been well studied. Mohavedi et al. (17) proposed three hypotheses to explain a surge in the incidence of DM during the COVID-19 pandemic. The first hypothesis is to accept it as true DM. There are known infectious agents which can trigger DM. Perhaps SARS-CoV-2 may be a more potent trigger for this disorder. The second is to consider it as prolonged post-viral myositis, which occurs following SARS-CoV-2 infections. Symptoms usually begin approximately 3–7 days after the onset of fever and respiratory symptoms and then resolve themselves within the 1st week but can persist for up to 1 month. Like other complications of COVID-19, this type of post-viral myositis may take a longer time for remission. Finally, it might be a DM-like syndrome, which mimics the symptoms of DM but is not real DM. DM-like syndrome can be another presentation of hyperinflammation syndromes due to COVID-19 (18).

In addition, De Santis et al. (19) analyzed the link between autoimmune diseases and environmental factors, in particular SARS-CoV-2 infection. This study demonstrated that viral infections such as COVID-19 are associated with ANA and specific autoantibodies directed toward antiviral signaling antigens, including autoantibodies specific for DM (De 19). Other reports have recently identified three immunogenetic epitopes, found in SARS-CoV-2-positive patients with autoimmune DM. It could suggest an overlapping mechanism for immune pathogenesis (20).

The hypothesis of SARS-CoV-2-triggering autoimmunity is gaining attention. In the “Molecular Immunology” journal, a study has identified 28 human proteins that contain regions homologous to SARS-CoV-2 peptides. These proteins could potentially act as autoantigens in COVID-19 patients with autoimmune conditions (17).

### 3.1. Literature review

In the literature, we came across case reports about COVID-related DM. According to Gokhale et al. (21), a surge in the incidence of DM was noted in 2020 during the months of April–August—the period coinciding with the occurrence of the COVID-19 pandemic in the City of Mumbai. Usually, they identified one to two patients of DM per year. At the time in question, the total number of cases encountered numbered five during a span of 6 months.

Similarly, in the pediatric population in Iran, between 2014 and 2019, two to four new cases of JDM were reported, while the last year from February 2020 to February 2021, eight new cases of JDM were reported (18).

In the “Rheumatology” journal, a 36-year-old woman with the onset of DM in close association with COVID-19 was described. Laboratory findings showed a positive pattern of ANA (1/640) and anti-Mi-2, with a significant increase in muscle enzymes (CK 3518 U/l) and specific findings in the muscle biopsy. The patient improved with a 5-day pulse of methylprednisolone (22). In addition, Tanboon et al. (23) commented on Zhang et al. (24) that a 58-year-old COVID-19-positive patient reported to have myositis may have had DM.

Undoubtedly, SARS-CoV-2 has an affinity for neural tissue (25), and neurological complications caused by COVID-19 are frequent (26). According to Mao et al. (27) in a Wuhan study, 36.4 % of infected patients exhibited a neurological manifestation. Apart from disorders of the central and peripheral nervous system, symptoms and disorders of muscular and neuromuscular transmission have also been described (28, 29), for instance, rhabdomyolysis (30), Guillain–Barré syndrome (GBS) (31), and other myositis (31). Skeletal muscle injury was reported in 19.3 % of patients (27), whereas, in another report, they were observed in 57.4 % of patients (32). The prevalence of myalgia varies between 11% and 50% (33).

Lehmann et al. (28) and Zheng et al. (29) provided an overview of frequently reported symptoms, such as myalgia as well as defined disorders, such as rhabdomyolysis, myositis, myasthenia, and intensive care unit (ICU)—acquired weakness, which has been described during COVID-19.

## 4. Summary

The association between COVID-19 and the development of multi-system autoimmune disorders remains unclear; however, viral infections such as SARS-CoV-2 may likely serve as a trigger. Further studies are needed to elucidate the pathomechanism, appropriate treatment, and long-term clinical outcomes of muscular manifestations associated with COVID-19 and to better understand the association of SARS-CoV-2 with DM. Our study has also a few methodological shortcomings to be improved in future. First, a baseline neurological examination is missing due to the patient reported to the neurological clinic after 6 months of illness, and previously, he had no physical examination by a neurologist. In addition, future studies should perform histopathological examinations showing feature characteristics of DM.

## Data availability statement

The original contributions presented in the study are included in the article/Supplementary material, further inquiries can be directed to the corresponding author.

## Ethics statement

The studies involving human participants were reviewed and approved by the Bioethics Committee of the Wroclaw Medical University (approval no. KB–552/2021). The patients/participants provided their written informed consent to participate in this study. Written informed consent was obtained from the individual(s) for the publication of any potentially identifiable images or data included in this article.

## Author contributions

JN: conceptualization, methodology, investigation, data curation, data analysis, original draft preparation, reviewing, and editing the manuscript. MC-M: conceptualization, methodology, reviewing, and editing the manuscript. JC-Ł: methodology, investigation, data curation, data analysis, original draft preparation, reviewing, and editing the manuscript. JB: methodology, data analysis, reviewing, and editing the manuscript. SK: conceptualization, methodology, data analysis, original draft preparation, reviewing, and editing the manuscript. PJ: conceptualization, methodology, investigation, data curation, data analysis, reviewing, and editing the manuscript. All authors have read and agreed to the published version of the manuscript.
