# Supplementary material for: Case report: Unusual patient with dermatomyositis associated with SARS-CoV-2 infection
Source: Front Neurol. 2023 May 19;14:1122475. doi: 10.3389/fneur.2023.1122475 (PMC10236949; doi:10.3389/fneur.2023.1122475)
Supplement: Supplementary file 1 [file Table_1.docx]

**SUPPLEMENTARY TABLE 1.** EMG findings.

| **Activity** | **Parameter** | **Tibialis anterior right** | **Vastus lateralis right** | **Biceps femoris right** |
| --- | --- | --- | --- | --- |
| Spontaneous activity | Fil | 3/10 | 4/10 | 3/10 |
|  | PSW | 3/10 | 4/10 | 3/10 |
|  | Amplitude | - | Normal | - |
| Voluntary activity | Dur | - | - | - |
|  | Poly | ++ | + | ++ |
|  | Stabil | Normal | Normal | Normal |
|  | IP | + | Normal | + |
|  | Rectrut | Early | - | Early |
| **Interpretation** | | **Myopathy** | **Myopathy** | **Myopathy** |
| MUP EMG | Dur [ms] | 11.3 | 10.4 | 7.6 |
|  | Amp [μV] | 508 | 843 | 414 |
|  | Poly [%] | 45.8 | 66.7 | 40.9 |
|  | Area [ms* μV] | 679 | 632 | 268 |
|  | Si [#] | 0.71 | 0.66 | -0.16 |
|  | Fr [Hz] | 7.1 | 10.0 | 12.5 |
